# *Plasmodium falciparum* diagnostic tools in HIV-positive under-5-year-olds in two ART clinics in Ghana: are there missed infections?

**DOI:** 10.1186/s12936-018-2231-7

**Published:** 2018-02-23

**Authors:** Ewurama D. A. Owusu, Samson K. Djonor, Charles A. Brown, Martin P. Grobusch, Petra F. Mens

**Affiliations:** 1 Division of Internal Medicine, Department of Infectious Diseases, Centre of Tropical Medicine and Travel Medicine, Academic Medical Centre, University of Amsterdam, Amsterdam, The Netherlands; 2 Department of Medical Laboratory Sciences, School of Biomedical and Allied Health Sciences, College of Health Sciences, University of Ghana, P.O. Box 0s123, Osu, Accra, Ghana; 3 Division of Laboratory Specialisms, Department of Medical Microbiology, Clinical Parasitology, Academic Medical Centre, University of Amsterdam, Amsterdam, The Netherlands; 4 Centre de Recherches Médicales de Lambaréné (CERMEL), Hôpital Albert Schweitzer, Lambaréné, Gabon; 5 Institute of Tropical Medicine, University of Tübingen, Tübingen, Germany

**Keywords:** Malaria, Microscopy, RDT, Diagnostic, Under-fives, HIV, Ghana

## Abstract

**Background:**

*Plasmodium falciparum,* the most dominant species in sub-Saharan Africa, causes the most severe clinical malaria manifestations. In resource-limited Ghana, where malaria and HIV geographically overlap, histidine-rich protein 2 (HRP2)-based rapid diagnostic test (RDT) is a faster, easier and cheaper alternative to clinical gold standard light microscopy. However, mutations in parasite *hrp2* gene may result in missed infections, which have severe implications for malaria control.

**Methods:**

The performance of a common HRP2-based RDT and expert light microscopy in HIV-positive and HIV-negative children under 5 years old was compared with PCR as laboratory gold standard. Finger-prick capillary blood was tested with First Response^®^ Malaria Ag *P. falciparum* (HRP2). Giemsa-stained thick and thin blood films were examined with ≥ 200 high power fields and parasites counted per 200 white blood cells. Nested PCR species identification of *P. falciparum* was performed and resolved on agarose gel. False negatives from RDT were further tested for deleted *pfhrp2/3* and flanking genes, using PCR. The study was performed in two anti-retroviral therapy clinics in Accra and Atibie.

**Results:**

Out of 401 participants enrolled, 150 were HIV positive and 251 HIV negative. Malaria was more prevalent in children without HIV. Microscopy had a higher sensitivity [100% (99–100)] than RDT [83% (53.5–100)]. Parasites with *pfhrp2/3* deletions contributed to missed infections from RDT false negatives.

**Conclusion:**

Circulation of malaria parasites with *pfrhp2/3* deletions in this population played a role in missed infections with RDT. This ought to be addressed if further strides in malaria control are to be made.

**Electronic supplementary material:**

The online version of this article (10.1186/s12936-018-2231-7) contains supplementary material, which is available to authorized users.

## Background

HIV and malaria geographically overlap in Ghana [1, 2]. HIV co-infection has been shown to increase the vulnerability of the population to malaria [3–5]. Out of the 9248 AIDS deaths reported in Ghana in 2014, 1295 (14%) were children; and 31.6% of the children who died from AIDS were under 5 years old. This age group is also considered a malaria-vulnerable group [6]. A mathematical model has been used to postulate that HIV increases the biomass of *Plasmodium* and results in an increase in emergence of anti-malarial resistance [7]. This was based on the assumption that biomass increment will increase the rate of mutations, if the probability of de novo resistance mutations is evenly distributed among all parasites [7]. Hence, drug administration will select for genetic mutations and spread drug resistance [7]. As such, a substantial contribution to malaria transmission and drug resistance might originate from this sub-population of patients living with HIV. It is imperative that missed malaria infections in this population are minimal, in order to control malaria transmission and reduce drug resistance.

Improved rapid diagnostic tests (RDT), which are faster, cheaper and easier compared to the gold standard expert microscopy, are complementing malaria control efforts in resource-limited sub-Saharan African countries such as Ghana [8]. In Ghana, most of the rapid diagnostic test kits that are available are based on histidine-rich protein-2 (HRP2) [9–11], which is an antigen exclusive to *P. falciparum* [12]. However, recent reports have shown that *hrp*-*2* deletions in parasite genes (*pfhrp2*), which previously prevailed only in the South Americas, have now been detected in African countries like Ghana, Democratic Republic of Congo, Eritrea, Uganda and Rwanda, regardless of level of transmission [8]. The lack of *pfhrp2* expression can prevent the detection of parasites by HRP2-based RDTs and give false negative results [8].

In spite of the preponderance of HRP2-based RDTs in Ghana, very limited studies have been conducted on the circulation of *pfhrp2* deletion in parasites in the Ghanaian population. An assessment of diagnostics in the Bongo District of northern Ghana showed that health workers in the Community-based Health Planning and Services (CHPS) had challenges with inaccurate or invalid RDT results, which were HRP2-based [13]. However, whilst there was no further investigation into possible circulation of parasites with *pfhrp2* gene deletions, another study found the mutation in Central (40%) and Greater Accra (22.4%) regions of Ghana [9]. Missed malaria infections, as a result of inability of RDTs to detect parasites, can result in increased morbidity and mortality, especially in children under 5 years old [8]. This could be more severe in people living with HIV. This study, therefore, looked at the performance of an HRP2-based RDT on the Ghanaian market and expert light microscopy, in under 5 year olds who are living with HIV in two anti-retroviral therapy (ART) centers in Atibie (Eastern region) and Accra (Greater Accra region), Ghana.

## Methods

### Study site and population

Data was collected at two ART clinics, the Ridge Regional Hospital and Kwahu Government Hospital. The Ridge Regional Hospital is located in the Greater Accra region whilst the Kwahu Government Hospital is located in the Eastern region of Ghana. Malaria prevalence in Greater Accra is relatively lower at 4–10%, whilst that in the Eastern region is relatively higher at 22% [10]. The vegetation in Eastern region is forest, whilst that in Greater Accra is coastal savannah. Malaria transmission is perennial in both study sites.

Both clinics provide health services to patients living with HIV. They also provide free access to anti-retroviral drugs, counseling and prophylaxis. HIV negative participants were recruited from the out-patient department (OPD) of the main hospital. For this category, only patients who had their HIV-negative status clearly indicated in their folders were included in this study.

### Study design and participant selection

This was a cross-sectional study, performed from May (beginning of the major raining season) to July 2015. The study was explained to parents of all children under 5 years old before enrollment. Consecutive patients attending the ART clinics (for HIV positive participants) and OPD of the main hospital (for HIV negative participants) were recruited into the study. Patient information such as age, sex, signs and symptoms were recorded. After malaria screening, participants were classified into asymptomatic or symptomatic groups. Those who had fever (axillary temperature ≥ 37.5 °C) with any of the following; chills and sweating, general malaise, headache, vomiting, nausea or diarrhea, were placed in the symptomatic category.

### Study procedure

#### Malaria screening

Finger-prick capillary blood of participants was taken for three different test procedures. Blood was dropped on First Response^®^ Malaria Ag *P. falciparum* (HRP2) malaria rapid diagnostic test kit and results recorded according to the instructions of the manufacturer. This RDT has passed the lot testing according to WHO-FIND guidelines, which subjects available test kits on the market to 3–6 rigorous rounds of testing [14]. One of the reasons why it is recommended for this country is because of its stability despite adverse weather conditions [14].

Thick and thin blood smears were prepared for malaria parasite detection and identification. Thin smears were fixed in 100% methanol after air-drying. Both were stained for 10 min with 10% Giemsa; and examined by two expert microscopists. Enumerating 200 high-powered visual fields, samples were recorded as positive if any amount of asexual parasites was observed per 200 white blood cells and negative if none was observed. A third microscopist resolved discordant results. Participants who tested positive for either RDT or blood smear were referred to attending doctor for appropriate treatment.

Three drops of blood were placed on filter paper (Whatman grade 3). These were dried, stored and later shipped for PCR analysis at the KIT Biomedical Research Institute in Amsterdam, the Netherlands. DNA was extracted using the Boom method [15] and nested PCR was performed for *P. falciparum* identification (see [16]). Samples that were RDT negative but PCR positive for *P. falciparum* were further tested for *pfhrp2/3* deletion, by amplifying the histidine-rich central repeat region using primers for the conserved 5′ and 3′ regions of *hrp2* and *hrp3* genes. The flanking genes for *hrp2* and *hrp3* were also amplified. A 25 µl total volume was used for PCR reactions, containing 5 µl DNA template, 10× buffer, 25 mM MgCl_2_, 10 mM each dNTP, 100 mM of each primer and 5 units of Taq (Roche diagnostics, Mannheim, Germany). Refer to Additional file 1 for primers used and PCR conditions. All PCR products were resolved on 2% agarose gel electrophoresis.

#### Statistical analysis

Data was entered in Microsoft Office Excel^®^ 2010 (Microsoft Corporation, USA), and statistically analysed with XLSTAT statistical ad-in. Diagnostic tools were compared using McNemar’s test. A p value ≤ 0.05 was considered as statistically significant. Kappa coefficients were used to test agreement between the diagnostic tools RDT and microscopy. Moderate agreement is indicated when kappa coefficient is between 0.4 and 0.6, substantial agreement when between 0.6 and 0.8 and excellent agreement when 0.8 or more [17].

The sensitivity and specificity of RDT and microscopy were calculated using PCR as the gold standard. The proportion of RDT and microscopy positives, individually, divided by the total positives determined by PCR was recorded as sensitivity. Specificity was calculated as the proportion of individual RDT and microscopy negatives divided by the total negatives in the PCR. The number of RDT (or microscopy) true positives divided by the number of all RDT (or microscopy) positive test results (true positive plus false positives) was recorded as positive predictive value (PPV); whilst negative predictive value (NPV) was recorded as the number of all RDT (or microscopy) true negatives, divided by all RDT (or microscopy) negative test results (true negatives plus false negatives).

## Results

### General study characteristics

A total of 150 HIV positive and 251 HIV negative children under 5 years old were recruited into the study. Male:female ratio was 19:131 in the HIV positive group, and 79:172 in the HIV negative group (Table 1). When RDT was used to test for malaria among both groups, 5/150 (3.3%) of HIV positive patients had malaria whilst 44/251 (17.5%) of those without HIV had malaria. Light microscopy and PCR gave similar results in patients living with HIV; 6/150 (4%) participants had malaria. However, amongst HIV negative participants, the results of these two diagnostics did not agree; 50/251 (19.9%) positives with light microscopy and 56/251 (22.3%) positives with PCR. Even though 60/150 (40%) of participants with HIV and 131/251 (52.2%) without HIV had malaria-suggestive symptoms of temperature, headache, vomiting and diarrhoea; malaria parasites were observed only in 6/150 (4%) with HIV and 56/251 (22.3%) without HIV using PCR (Table 1).

**Table 1:** Socio-demographic characteristics and malaria diagnosis of under 5 year olds with HIV and without HIV

Category	Variable	Frequency (%)	
		HIV+	HIV−
Age	Under 5	150 (100%)	251 (100%)
Sex	Male	19 (12.7%)	79 (31.5%)
	Female	131 (87%)	172 (68.5%)
RDT diagnostics	Negative	145 (96.7%)	207 (82.5%)
	Positive	5 (3.3%)	44 (17.5%)
Light microscopy	Negative	144 (96%)	201 (80.1%)
	Positive	6 (4%)	50 (19.9%)
	Mean parasite density/µl (CI)^a^	80,400 (19,201–141,559)	89,700 (53,121–126,279)
PCR	Negative	144 (96%)	195 (77.7%)
	Positive	6 (4%)	56 (22.3%)
Malaria-like symptoms	No	90 (60%)	120 (47.8%)
	Yes	60 (40%)	131 (52.2%)

^a^Mean of those who tested positive

Based on microscopy, the mean parasitaemia of all patients was 85,050/µl. When the groups were separated into HIV positive and HIV negative, the mean parasitaemia were 80,400/µl (19,201–141,559) and 89,700 (53,121–126,279), respectively.

### Comparison of diagnostic tools and accuracy tests

There were significant differences between the performances of RDT, microscopy and PCR (p < 0.05) (Table 2). Yet, Kappa coefficient to determine agreement between RDT and microscopy showed excellent agreement at 0.83. In general, the sensitivity for expert light microscopy was higher [100% (99–100) and 89.3% (81.2–97.4)] than RDT [83% (53.5–100) and 78.6% (67.8–89.3)] in HIV-positive and -negative participants, respectively (Table 2). Specificity of RDT for both HIV-positives and -negatives were 100% (98.9–100 and 100% (99.1–100). The positive predictive values (PPV) were 100% (97.8–100) and 100% (99.3–100) in HIV positive and negative populations when RDT was used. Microscopy also yielded 100% (99.2–100) and 100% (99–100) positive predictive values. The negative predictive values for RDT were 99% (98–100) and 94.2% (91–97.4) in HIV positive and negative individuals, respectively. Negative predictive values were higher with microscopy; 100% (99.6–100) among HIV positives and 97% (94.7–99.4) among HIV negatives.

**Table 2:** A comparison of the diagnostic tools RDT, microscopy and PCR; and test of their accuracy

	RDT/PCR	Microscopy/PCR	RDT/microscopy
Neg	352/339	345/339	352/345
Pos	49/62	56/62	49/56
p value*	< 0.05	< 0.05	< 0.05
		Kappa co-efficient	0.83

RDT	Under 5 years (n = 150)	Under 5 years (n = 251)	
	HIV positive (95% CI)	HIV negative (95% CI)	
Sensitivity	83 (53.5–100)	78.6 (67.8–89.3)	
Specificity	100 (98.9–100)	100 (99.1–100)	
Positive predictive value	100 (97.8–100)	100 (99.3–100)	
Negative predictive value	99 (98–100)	94.2 (91–97.4)	

Microscopy	Under 5 years (n = 150)	Under 5 years (n = 251)	
	HIV positive (95% CI)	HIV negative (95% CI)	
Sensitivity (CI)	100 (99–100)	89.3 (81.2–97.4)	
Specificity (CI)	100 (98.5–100)	100 (99.3–100)	
Positive predictive value	100 (99.2–100)	100 (99–100)	
Negative predictive value	100 (99.6–100)	97 (94.7–99.4)	

* p value using McNemar’s test

All participants living with HIV who tested positive with RDT (5/5) were true positives if PCR was used as the gold standard (Table 3). In the HIV-negative group, all malaria positives (44/44) obtained with RDT were true positives. There were no false positives in both HIV negative and HIV positive participants. However, there were false negatives; 1/145 negatives in those with HIV and 12/207 in HIV negatives. Minimum parasite densities of the false negatives were 17,087 and 11,107 p/µl, respectively, in HIV-positive and HIV-negative groups.

**Table 3:** The differences in parasite detection amongst the two methods, using PCR as the gold standard

	Variable	HIV+	Minimum parasite density/µl	HIV−	Minimum parasite density/µl
RDT^a^	True positive	5	19,608	44	53,846
	False positive	0	–	0	–
	Total test positive	5	19,608	44	53,846
	False negative	1	17,087	12	11,107
	True negative	144	–	195	–
	Total test negative	145		207	
Microscopy	True positive	6	19,201	50	53,121
	False positive	0	–	0	–
	Total test positive	6	19,201	50	53,121
	False negative	0	–	6	21
	True negative	144	–	195	–
	Total test negative	144		201	
PCR	Gold standard	6	19,608	56	53,846

^a^Parasite count for RDT was done with real time PCR

Further test was done with PCR to detect *pfhrp2* and *pfhrp3* and their flanking genes in the RDT false negatives. However, there was not enough extracted DNA in 5 out of the 13 false negatives to perform these tests on. The *pfrp2/3* deletion tests on the remaining eight showed that the *pfhrp2* gene was present in 2 out of the 8 samples, one of which also had the flanking gene MAL7P1.228. One other sample had only MAL7P1.228 gene (12.5%) (Table 4). None had the *pfhrp3* gene nor its flanking genes.

**Table 4:** Pattern of deletion of *pfhrp2*, *pfhrp3* and their flanking genes in RDT false negatives

Pfhrp2 exon 2, PF3D7_0831800	Pfhrp2 exon 1–2, PFD7_0831800	PFD7_0831900 (MAL7P1.230)	PFD7_0831700 (MAL7P1.228)	Pfhrp3 exon 1–2, PF3D7_1372200	PF3D7_1372400 (MAL13P1.475)	PF3D7_1372100 (MAL13P1.485)	Study population	Frequency (%)
+	+	+	−	−	−	−	HIV negative	1/8 (12.5)
+	+	−	−	−	−	−	HIV negative	1/8 (12.5)
−	−	+	−	−	−	−	HIV negative	1/8 (12.5)
−	−	−	−	−	−	−	Both HIV negative and positive	5/8 (62.5)
+	+	+	+	+	+	+	Positive control	–
−	−	−	−	−	−	−	Negative control	–

## Discussion

It has been well established before that malaria and HIV interact manifold with each other [1–4]. The study population for this study had been chosen in the context of improving care for under-5-year-old HIV patients in whom, as with all children in endemic areas, malaria continues to constitute a major health threat and frequent cause of fever. The problem identified and discussed here, namely *pfhrp2, 3* depletions leading to false negative malaria RDT results, is not considered specific to the HIV-positive population, or the particular young age group.

This study has demonstrated that the sensitivity and specificity of both HRP2-based RDT and expert light microscopy are generally high in rural and urban Ghana when tested in children with HIV under 5 years old; and microscopy more so than RDT. Missed infections from microscopy were few, but generally higher in rural Atibie (Kwahu). The HRP2-based RDT used in this study missed 1/6 malaria infections in HIV positive and 12/56 in HIV negative participants. The parasite densities of the missed infections were high (minimum of 11,107 p/µl); therefore, low parasitaemia could not have been the reason for lack of detection with RDT. Further PCR test of these false negatives for *pfhrp2*, *pfhrp3* and their flanking genes indicated the presence of gene deletions in circulation.

Missed infections from microscopy were fewer than those from RDT. The false negatives from microscopy were higher in rural Atibie than in urban Accra. This might be attributed to the subjectivity of expert microscopy to individual bias. However, state-of-the-art RDTs should be able to rectify that; but false negatives were observed in the HRP2-based RDT used. The circulation of *pfhrp2* deleted malaria parasites has been established as one of the possible reasons for false negatives in RDTs in malaria-endemic areas. One of the very few prior studies done in Ghana has shown the deletions to be present in parasites in Accra and Cape-Coast [9]; whereas in other West African countries, several studies have found them to abound [8, 18, 19]. In this study, they were found to circulate in both the rural highland and the urban lowland. The circulation of these mutants in the population has dire implications for malaria control in Ghana. Among the malaria-vulnerable population of people living with HIV, the consequence of missed infections may be more serious; and even exceedingly so when they are under 5 years old. As has been established in the literature, malaria morbidity and mortality is worse in children under 5 years old [8, 20–22] and in people living with HIV [3, 23]; a combination of these two factors is even more severe [3, 4]. It is, therefore, imperative that alternative RDTs are used in Ghana, a good option will be ones which detect non-HRP2 antigens. However, those that are currently WHO pre-qualified or meet the WHO recommended procurement criteria are insufficient [8]. The development of RDTs that detect a combination of antigens might also be beneficial, so that mutant parasites with deletions will not be missed.

The contribution of *Plasmodium* parasitaemia in people living with HIV to anti-malarial drug emergence has been shown in other studies [7]. Perhaps low malaria prevalence, as found in this HIV population under 5 years of age, might not influence parasite biomass and contribute to de novo resistance mutations. Yet, rather than use parasite count of peripheral blood as an indication of parasite biomass since it does not take into consideration sequestered parasite load, there have been suggestions to use plasma *Pfhrp2* concentrations as estimates [24]. However, in the light of the circulation of *Pfhrp2* deletions in this study and others, this might not be an accurate estimation after all.

In a study in other parts of Ghana by Adu-Gyasi et al. [25], malaria prevalence in HIV positive patients was lower than the average national malaria prevalence. This is similar to the present study results; and may be attributed to co-trimoxazole (CTX) prophylaxis (given with anti-retroviral therapy as recommended by the Ghana National Aids Control Programme) [26]. All the under-5-year-olds living with HIV in this study were on prophylactic CTX. This has been shown to reduce opportunistic infections and protozoan infections like malaria [27–29].

Even though 40% of the children living with HIV in this study had symptoms that were indicative of malaria, such as fever, chills and sweating, headache, general malaise, vomiting, diarrhea or nausea, PCR showed that only 4% of that category actually had malaria. In a similar study in people living with HIV in Brong-Ahafo and Ashanti regions in Ghana, 38.1% of study participants had malaria symptoms yet only 4.4% were laboratory confirmed [25]. This suggests that the adoption of the WHO recommendation for laboratory diagnosis of malaria instead of the erstwhile clinical diagnosis by Ghana is a step in the right direction. It will reduce prescription of anti-malarials to only when it is necessary.

Ideally, subject to financing, an expansion of this study to other regions in Ghana should reveal the pattern of distribution of the *pfhrp2* mutants. Nonetheless, this is one of the first studies in Ghana that has looked at the performance of popular diagnostic tools in a malaria vulnerable population with the added vulnerability of HIV. It has also detected the circulation of parasites with *pfhrp2* deletions in this malaria-vulnerable population in the rural highland and urban lowland. This has shed light on the malaria situation of the malaria vulnerable sub-population and may contribute to adaptation of improved control measures.

## Conclusion

Malaria efforts in Ghana are commendable, yet improved focus on malaria vulnerable populations such as children under 5 years old living with HIV is necessary. Current diagnostic tools used, HRP2-based RDT and expert microscopy, have high sensitivities and specificities. Nonetheless, missed infections due to the presence of parasites with deleted *pfhrp2* genes need to be addressed if further strides towards malaria elimination are to be made.

## Additional file

**Additional file 1.** PCR reaction conditions and primer sequences. The conditions and primer sequences for amplification of *pfhrp2*, *pfhrp3* and their flanking genes were adapted from Abdallah et al. [30].
