# Supplementary material for: Plasmodium falciparum diagnostic tools in HIV-positive under-5-year-olds in two ART clinics in Ghana: are there missed infections?
Source: Malar J. 2018 Feb 23;17:92. doi: 10.1186/s12936-018-2231-7 (PMC5824602; doi:10.1186/s12936-018-2231-7)
Supplement: Supplementary file 1 — Additional file 1. PCR reaction conditions and primer sequences. The conditions and primer sequences for amplification of pfhrp2, pfhrp3 and their flanking genes were adapted from Abdallah et al. [30]. [file 12936_2018_2231_MOESM1_ESM.docx]

Additional file 1

PCR reaction conditions and primer sequences.

The conditions and primer sequences for amplification of pfhrp2, pfhrp3 and their flanking genes were adapted from Abdallah *et al*., 2015.

| **Gene** | **Reaction** | **Primer name** | **Primer sequence** | **Annealing temperature ( ◦C)** | **Expected amplicon size (bp)** |
| --- | --- | --- | --- | --- | --- |
| Pfhrp2 exon 1-2Pf3D7_0831800 | Primary | 2E12F1 | 5′ GGT TTC CTT CTC AAA AAA TAA AG 3′ | 55 | 228 |
|  |  | 2E12R1 | 5′ TCT ACA TGT GCT TGA GTT TCG 3′ |  |  |
|  | Nested | 2E12F | 5′ GTA TTA TCC GCT GCC GTT TTT GCC 3′ | 62 | 198 |
|  |  | 2E12R1 | 5′ CTA CAC AAG TTA TTA TTA AAT GCG GAA 3′ | |  |
|  |  |  |  |  |  |
| Pf3D7_0831900 (MAL7P1.230) | Primary | 230F1 | 5′ GAT ATC ATT AGA AAA CAA GAG CTT AG 3′ | 63 | 301 |
|  |  | 230R | 5′ TAT CCA ATC CTT CCT TTG CAA CAC C 3′ | |  |
|  | Nested | 230F1 | 5′ TAT GAA CGC AAT TTA AGT GAG GCA G 3′ | 65 | 228 |
|  |  | 230R | 5′ TAT CCA ATC CTT CCT TTG CAA CAC C 3′ | |  |
|  |  |  |  |  |  |
| PF3D7_0831700 (MAL7P1.228) | Primary | 228F | 5′ AGA CAA GCT ACC AAA GAT GCA GGT G 3′ | 60 | 198 |
|  |  | 228R | 5′ TAA ATG TGT ATC TCC TGA GGT AGC 3′ |  |  |
|  | Nested | 228F1 | 5′ CCA TTG CTG GTT TAA ATG TTT TAA G 3′ | 63 | 241 |
|  |  | 228R | 5′ TAA ATG TGT ATC TCC TGA GGT AGC 3′ |  |  |
|  |  |  |  |  |  |
| PfHRP3 Exon 1–2, PF3D7_1372200 | Primary | 3E12F1 | 5′ GGT TTC CTT CTC AAA AAA TAA AA 3′ | 53 | 225 |
|  |  | 3E12R1 | 5′ CCT GCA TGT GCT TGA CTT TA 3′ |  |  |
|  | Nested | 3E12F | 5′ ATA TTA TCG CTG CCG TTT TTG CT 3′ | 62 |  |
|  |  | 3E12R | 5′ CTA AAC AAG TTA TTG TTA AAT TCG GAG 3′ | |  |
|  |  |  |  |  |  |
| PF3D7_1372400 (MAL13P1.475) | Primary | 475F | 5′ TTC ATG AGT AGA TGT CCT AGG AG 3′ | 55 | 212 |
|  |  | 475R | 5′ TCG TAC AAT TCA TCA TAC TCA CC 3′ |  |  |
|  | Nested | 475F | 5′ TTC ATG AGT AGA TGT CCT AGG AG 3′ | 61 |  |
|  |  | 475R1 | 5′ GGA TGT TTC GAC ATT TTC GTC G 3′ |  |  |
|  |  |  |  |  |  |
| PF3D7_1372100, (MAL13P1.485 | Primary | 485F | 5′ TTG AGT GCA ATG ATG AGT GGA G 3′ | 60 | 241 |
|  |  | 485R | 5′ AAA TCA TTT CCT TTT ACA CTA GTG C 3′ | |  |
|  | Nested | 485F1 | 5′ GTT ACT ACA TTA GTG ATG CAT TC 3′ | 59 |  |
|  |  | 485R | 5′ AAA TCA TTT CCT TTT ACA CTA GTG C 3′ | |  |
